# Repetitive transcranial magnetic stimulation for post stroke rehabilitation—a single center community experience

**DOI:** 10.3389/fneur.2026.1792409

**Published:** 2026-03-18

**Authors:** Francois Henri Jacques, Johanna Acosta Diaz, Emilie Deschenes, Nassima Rabia, Bradley Erik Apedaile

**Affiliations:** 1 Clinique Neuro-Outaouais, Gatineau, QC, Canada; 2 Clinique NeuroGym, Gatineau, QC, Canada

**Keywords:** chronic stroke, neuronavigation, physiotherapy, post stroke rehabilitation, rTMS, stimulation protocol, stroke

## Abstract

Repetitive transcranial magnetic stimulation (rTMS), a form of non-invasive neuromodulation, has been shown to improve recovery post stroke. The definitive mechanism by which it alleviates symptoms remains elusive and as such many questions as to how to predict and improve clinical response are left unanswered. We conducted a retrospective chart review of our experience using rTMS combined with physiotherapy in post stroke patients at our clinic between 2014 and 2025. We reviewed 35 charts and found 23 that qualified. The mean age was 59.6 years, 57 percent male. Overall, 70 percent improved significantly in one or more measurements. Patients who received bilateral stimulation were 2.8 (95 percent confidence interval 1.4–7.8) times more likely to improve compared to patients who received unilateral stimulation. The mean time from the stroke to treatment in the improved group was 13.1 (4–35) months while in the unimproved group was 33.3 (5–80) months (*p* = 0.027). We found that rTMS combined with physiotherapy is effective in chronic post stroke patients. Time between the stroke and the beginning of treatment is inversely related to rTMS response and bilateral stimulation is more effective than unilateral stimulation.

## 1 Introduction

Globally, stroke is the leading cause of neurological disability and a significant financial burden to families and to the health systems (1). Only a minority of stroke patients recover full motor function (2). Repetitive transcranial magnetic stimulation (rTMS) is a form of non-invasive neuromodulation (NINM) whereby an electromagnet placed over the scalp produces repetitive, brief, magnetic pulses inducing focal currents in the underlying cortex (3). rTMS has been studied in a variety of psychiatric and neurological conditions including post stroke recovery (4–7). In several clinical trials, rTMS has been shown to improve post stroke recovery, including improvement in strength, swallowing, speech, walking, depression and neurogenic pain (8–10). The interhemispheric inhibition (11) and compensation models (12) represent some of the theoretical basis for rTMS therapy in post stroke rehabilitation. rTMS has also been shown to influence neurotransmitter (glutamate, gaba, acetylcholine) levels (13, 14), polarization of immune cells such as astrocytes (15) and microglia (9, 16) and shifting of cytokine production to a non-inflammatory profile (17, 18). Proposed neurophysiological mechanisms include increasing cortical excitability, restoring interhemispheric balance and improving functional connectivity and neuronal network organization. rTMS also improves neuroplasticity through facilitation of neural connection, promotion of synaptic strength and activity dependant myelination. However, the underlying mechanism by which rTMS helps to alleviate symptoms in a variety of conditions remains unknown.

There have been several different rTMS stimulation protocols used in clinical trials that have been shown, to varying degrees, to benefit post stroke recovery, including unilateral versus bilateral stimulation, low frequency versus high frequency stimulation, traditional versus theta burst, one site versus multiple sites, number of stimulations per session and number of total sessions (8–10, 19–22). Definitive conclusions from the clinical trials have been limited by small sample sizes, short duration, lack of control groups, accompanied or not by concurrent adjunctive therapies such as physiotherapy (8, 9). Some of the questions that remain are which is the best rTMS stimulation protocol and for which symptoms, what is the ideal number of stimulations per session and total number of sessions, is maintenance therapy indicated, and what are the patient prognostic factors that would predict higher likelihood of response to rTMS. Our center has offered combined rTMS and physiotherapy for more than 10 years. Our rTMS treatment approach has evolved through a trial-and-error design. We have reviewed our experience to answer some of the unanswered questions.

## 2 Methods

A retrospective chart review was performed of patients who received rTMS for post stroke rehabilitation at our clinic in Gatineau, Canada between 2014 and 2025. Only patients with objective pre and post rTMS measurements of clinical improvement were included. Demographic and efficacy data and stimulation protocols were collected and analyzed. Relative risk was calculated to compare the probability of improving with bilateral stimulation compared to unilateral stimulation. A two-sample *t*-test was used to compare the mean age of Protocol A (unilateral stimulation) with the mean age of Protocols B and C combined (bilateral stimulation) and a 2-sample test for equality of proportions was used to compare the proportion of males in Protocol A with the proportion of males in the combined Protocols B and C. Results are provided in Table 1. All statistical analysis was performed using R v 4.5.2. (23).

**Table 1:** Patient demographics.

Variable	All	Protocol A	Protocol B	Protocol C	*p* value^1^
Age, mean years (range)	59.6 (29–82)	55.8 (39–75)	67.5 (56–82)	59.9 (29–77)	0.244^2^
Male, *n* (%)	13 (57%)	7 (88%)	2 (50%)	4 (40%)	0.085^3^
Time since stroke, mean months (range)	19.2 (4–80)	25.9 (4–72)	14.3 (5–29)	13. (1–34)	0.143^2^
Hemorrhagic stroke, *n* (%)	3 (13%)	1 (11%)	0 (0%)	2 (20%)	
Ischemic stroke, *n* (%)	20 (87%)	8 (89%)	4 (100%)	8 (80%)	
Secondary epilepsy, *n* (%)	6 (26%)	4 (44%)	0 (0%)	2 (20%)	
Depression, *n* (%)	2 (9%)	1 (11%)	0 (0%)	1 (10%)	
Neurogenic pain, *n* (%)	5 (22%)	3 (33%)	1 (25%)	1 (10%)	
Aphasia *n* (%)	4 (17%)	1 (11%)	0 (0%)	3 (30%)	

1 Comparing protocols B and C (bilateral stimulation) with Protocol A (unilateral stimulation).

2 Welch two sample *t*-test.

3 2-sample test for equality of proportions.

Ethical approval was granted by the Canadian SHIELD Ethics Review Board (approved October 23, 2023; Principal Investigator: Dr. Jacques). Consent materials were available in both English and French. A note to file was received by Canadian Shield for patients who received their treatments prior to 2023 agreeing to the use of their anonymized data in our research.

A Magstim Rapid 2 (Magstim Whitland, U.K.) with the D70A butterfly and the double cone (DCC) coils were used for stimulation. Protocols had intensity ranging from 110 percent of resting motor threshold to 100 percent of machine output. A neuro-navigation system (Rogue Research, Montreal, Canada) was used to identify the stimulation sites and to orient the coils. A Natus Nicolet Viking EDX EMG machine (Natus Medical Inc. Middleton WI, USA) was used to determine the resting motor threshold.

Improvement was defined as an increase in grip strength of 50 percent, a reduction in the 9-hole peg test (9HPT) time of 20 percent, a reduction in the 10 m walk time (10MWT) of 20 percent, an increase in the 6 min walk test (6MWT) of 50 m or 50 percent, or an increase in the Fugl- Meyer Assessment Upper Extremity score of 10 points, compared to the pre rTMS baseline. Subjective improvements in post stroke depression and pain were also noted.

Over an 11-year period, rTMS protocols were modified to improve efficacy and tolerance. They were classified into three approaches that evolved over time from initial to most recent. Protocol A used a frequency of 1 Hz with intensity at 100 percent to 110 percent of resting motor threshold, unilateral stimulation of the M1 hand area of the unaffected hemisphere. Protocol B started with protocol A and then after 10–20 sessions, if the response was inadequate, switched to bilateral stimulation, including the unaffected hemisphere M1 hand area and the affected hemisphere perilesional area. Protocol C used a frequency of 1 Hz, bilateral stimulation using four different targets (unaffected hemisphere M1 hand area, affected hemisphere perilesional hand area, Cz and Pz) and adjusting the number of stimulations and intensity, aiming for maximum machine output and approximately 2000 stimulations per session according to patient tolerance.

Additional details of the protocols are available in the Supplementary material. All patients received 30 or more consecutive, daily (Monday–Friday) sessions of rTMS followed by physiotherapy.

## 3 Results

**Figure 1:**
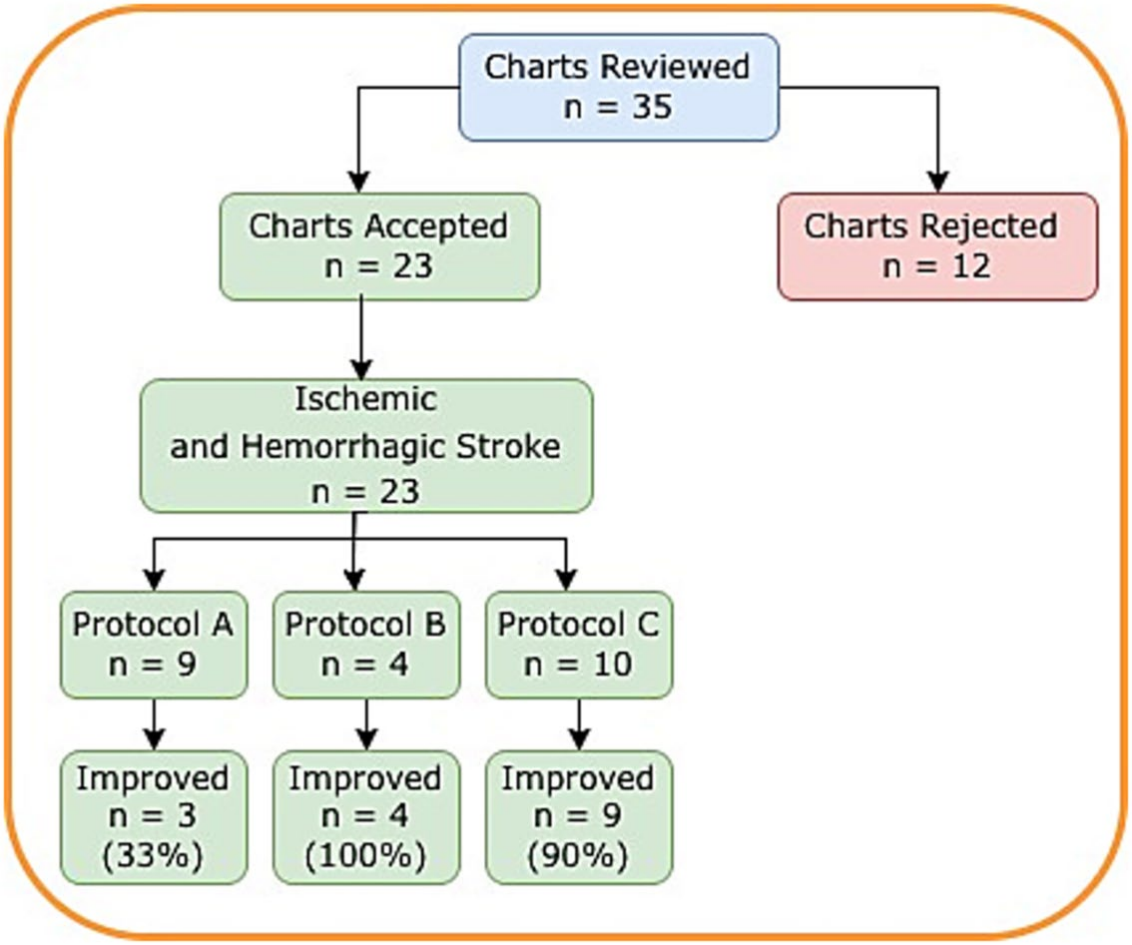
Disposition of patients.

Thirty-five patient charts were reviewed. Twenty-three patients had documented pre and post objective measures of physical disability. Nine patients were treated using protocol A, four using protocol B and 10 using protocol C (Figure 1).

The mean age of the patients was 59.6 (29–82) years and 57 percent (*n* = 13) were male (Table 1). In 20 patients, the stroke was ischemic and in three patients hemorrhagic. Six patients had secondary epilepsy, two had depression, five had neurogenic pain, and four had variable degrees of aphasia. The mean time elapsed from stroke to rTMS therapy initiation was 19.2 (4–80) months. Only four patients were less than 6 months.

Overall, 70 percent (*n* = 16) showed improvement from pre rTMS in at least one measurement. Thirty-nine percent (*n* = 9) improved in one criterion, 26 percent (*n* = 6) in two criteria and 4 percent (*n* = 1) in three criteria. Under protocol A, 33 percent (*n* = 3) improved, protocol B, 100 percent (*n* = 4) improved and protocol C, 90 percent (*n* = 9) improved. Patients given bilateral treatment were 2.8 times (1.4–7.8) more likely to see an improvement compared to patients receiving unilateral treatment.

Those who improved also tended to have had less time elapsed between their stroke and rTMS treatment initiation. The mean time from the stroke to treatment in the improved group was 13.4 (4–34) months while in the unimproved group was 31.6 (4–72) months (*p* = 0.027). All patients with post stroke pain and/or depression showed significant improvement or complete resolution following rTMS therapy. Of the six patients with secondary epilepsy at baseline, none suffered a seizure over the course of the rTMS treatments.

## 4 Discussion

Our chart review, though uncontrolled, adds to the growing evidence that rTMS combined with physiotherapy can be effective in improving post stroke motor recovery in patients with chronic strokes (more than 6 months). It aligns with previous research demonstrating that a longer time between stroke occurrence and the beginning of treatment is a negative prognostic predictor of rTMS response. Three meta-analyses by Zhang et al. (9), Fan et al. (10) and Alhalabi et al. and the 2019 guidelines of the International Federation of Clinical Neurophysiology (24) all suggest that rTMS efficacy is time dependant with the best chances of response occurring in the acute/subacute stages of recovery post stroke. The latter is corroborated by research suggesting that altered mechanisms of neuroplasticity in post stroke patients are also time dependant (25). Our use of 30 sessions is underlaid by Alhalabi et al.’s meta-analysis of rTMS for upper limb recovery in post stroke patients which suggests that 20 sessions are more efficacious than 10 sessions (19). Our use of combined neurorehabilitation and rTMS is supported by Stewart and Alabbas, in their review of rTMS in post stroke motor recovery which concluded that early rehabilitation plays an important role in post stroke recovery and that rTMS can increase the rate and quality of functional improvement (26) and by Yamamoto et al.’s 4 week, three-arm randomized control trial which concluded that the addition of rTMS to neurorehabilitation improved post stroke recovery when compared to neurorehabilitation (21).

In our chart review of mostly chronic patients, bilateral stimulation was more effective than traditional unilateral stimulation of the unaffected hemisphere. Alhalabi et al. showed that stimulation of the affected hemisphere was more effective than stimulation of the unaffected hemisphere (19), while Zhang et al. suggested that bilateral stimulation had a more sustained improvement than unilateral stimulation of either the affected or unaffected hemisphere (9). Fan et al. concludes that no definitive evidence exists to designate which approach is best (10). In the reviews by Zhang (9), Fan (10) and Sheng (8), the optimal parameters for stimulation frequency and intensity remain unanswered. Du et al.’s sham-controlled study showed that either high frequency stimulation to the affected hemisphere or low frequency stimulation to the unaffected hemisphere resulted in greater motor recovery than sham treatment (27). The discordant results regarding frequency can be explained by Prei et al.’s research which demonstrated a lack of evidence supporting the heuristic thinking that low frequency inhibits and high frequency excites in healthy controls (28). At our center, because of the positive results for both low and high frequency rTMS stimulation protocols we have migrated to using only 1 Hz, allowing the use of the highest stimulation intensity tolerated by the patient, which ranged from 60 to 100 percent of machine output depending on the target. It is our opinion that since penetration and resulting strength of the induced current is proportional to the strength of the magnetic field, especially when stimulating the perilesional cortex, a stronger induced cortical current is more likely to reach cortical tissue relevant to neuroplasticity and have a greater impact on cortical excitability, immune cells, neurotransmitters and cytokine production (8).

Neuroimaging studies suggest that motor disability after a stroke is the result of the disturbance of the entire motor network rather than the removal of a single item within the network. Post stroke functional imaging will initially be characterized by a reduction in the functional connectivity between hemispheres especially the homotopic area of the damaged region and in the effective connectivity between different areas within the affected hemisphere connected to the damaged region. Motor recovery is corroborated by an improvement in both the interhemispheric and intrahemispheric connectivity. The beneficial effect of rTMS may be its ability to restore the connectivity between the stimulation site and remote motor areas (27, 29). We propose that bihemispheric stimulation is more effective than unilateral stimulation because of its greater likelihood of modulating the cortical excitability of the perilesional intact cortex by stimulating it directly using neuronavigational targeted stimulation and through its homotopic area via the corpus callosum. Bilateral stimulation through multiple motor sites including connectome hubs within the motor system intuitively has a greater probability of reorganizing neuronal networks and facilitating neuroplastic changes through activity dependant myelination, increased synaptic strength and facilitating new neural connections than unilateral, single site stimulation.

Our study is limited by its retrospective nature, small sample size, lack of controls, no long-term follow-up and our inability to distinguish the benefits obtained from physiotherapy from those induced by the rTMS. Some of the limitations should be nuanced by the fact that the mean time from stroke occurrence was 21 months, making spontaneous recovery very unlikely.

## 5 Conclusion

rTMS in a clinical setting, is effective when combined with physiotherapy in improving motor recovery in chronic (more than 6 months) post stroke patients. Time between the stroke and the beginning of treatment is inversely related to rTMS response. Bilateral stimulation is more effective than unilateral stimulation. Many questions about the optimal rTMS treatment paradigm remain unanswered.

## Data Availability

The raw data supporting the conclusions of this article will be made available by the authors, without undue reservation.
