# Supplementary material for: Repetitive transcranial magnetic stimulation for post stroke rehabilitation—a single center community experience
Source: Front Neurol. 2026 Mar 18;17:1792409. doi: 10.3389/fneur.2026.1792409 (PMC13040469; doi:10.3389/fneur.2026.1792409)
Supplement: Supplementary file 1 [file Table_1.DOCX]

Supplementary Material

**RTMS Methods**

A Magstim Rapid 2 (Magstim Whitland,U.K.) with the D70A butterfly and the double cone (DCC) coils were used for stimulation. The electric and magnetic fields produced according to distance from the coil surface at 100% maximum machine output are shown in Table 1.

Table 1 MagStim Rapid-2 TMS E-Field and Bx-Field at Maximum Output

| Distance from magnet surface | E field^1^ DCC^2^ | E field D70A^3^ | Bx field DCC^4^ | Bx field D70A |
| --- | --- | --- | --- | --- |
|  | (V/m) | (V/m) | (Tesla) | (Tesla) |
| 1cm | 331 | 175 | 0.65 | 0.65 |
| 2cm | 242 | 111 | 0.50 | 0.40 |
| 3cm | 174 | 71 | 0.39 | 0.26 |
| 4cm | 123 | 45 | 0.31 | 0.18 |
| 5cm | 84 | 29 | 0.24 | 0.13 |
| 6cm | 55 | 18 | 0.18 | 0.09 |
| 7cm | 35 | 11 | 0.15 | 0.07 |
| 8cm | 21 | 6 | 0.11 | 0.06 |

^1^ E field – Electric Field

^2^DCC - Double Cone Coil

^3^D70A – Butterfly Coil

A neuro-navigation system (Rogue Research, Montreal, Canada) was used to identify the stimulation sites and to orient the coils. A Natus Nicolet Viking EDX EMG machine (Natus Medical Inc. Middleton WI, USA) was used to determine the resting motor threshold.

**Resting Motor Threshold Procedure**

The motor threshold is determined to individualize stimulation intensity for rTMS treatment. It represents the minimum stimulation intensity required to elicit a motor response in a target muscle.

1. Localization of Motor Cortex

- The coil is positioned over the hand area of the primary motor cortex (M1) using the neuronavigation system identifying the Pli de Passage Moyen.
- Coil orientation is adjusted to maximize motor-evoked responses in the ADM muscle.

1. Initial Stimulation

- Single-pulse TMS is delivered at 100% intensity and is gradually adjusted to identify the lowest level that produces a visible or measurable motor response.

1. Response Criteria

- A visible twitch of the ADM muscle
- An EMG response of ≥50 µV peak-to-peak amplitude obtained at least 50% of the time

The procedure is performed separately for each hemisphere, as thresholds may differ.

**RTMS stimulation protocols**

**Protocol A**

The M1 hand area of the unaffected hemisphere was the stimulation target.

100 -110% of the resting motor threshold was used as the stimulation intensity at 1 hz stimulation frequency.

1500 stimulations were given per session.

The sessions were administered daily except for weekends, and a treatment cycle consisted of 30 sessions.

All sites are determined by neuronavigation and adjusted slightly for best response (hot spot)

**Protocol B**

The treatment cycle is initiated using protocol A.

After 10 to 20 sessions if no significant improvement is seen then the protocol is changed to a bilateral approach consisting of stimulation of the M1 hand area of the unaffected hemisphere using 1hz and 500 stimulations per session and the perilesional area of the M1 hand area of the affected hemisphere using 1 hz and 1000-1500 stimulations were administered per session. The sessions were administered daily except for weekends, and a treatment cycle consisted of 30 sessions.

All sites are determined by neuronavigation and adjusted slightly for best response (hot spot)

**Protocol C**

The stimulation targets are the M1 of the hand area in the unaffected hemisphere, the perilesional cortex of the M1 hand area of the affected hemisphere, the Cz and Pz (10-20 EEG measurement system). All sites are determined by neuronavigation and adjusted slightly for best response (hot spot)

A butterfly coil (D70A) was used for the hand area and a double cone coil (DCC) for Cz and Pz targets.

A 1hz stimulation frequency is used and a starting intensity of 60-70% of machine output and 500 stimulations per site for a total of 2000 stimulations per session.

The intensity and relative number of stimulations per site is adjusted according to tolerance and response. The number of stimulations per session ranged from 2000- 2500.

The sessions were administered daily except for weekends, and a treatment cycle consisted of 30 sessions.
